# Oxidative Stress–Telomere Axis in IVF: Molecular Mechanisms, Biomarkers, and Clinical Translation

**DOI:** 10.3390/ijms262311359

**Published:** 2025-11-24

**Authors:** Charalampos Voros, Fotios Chatzinikolaou, Georgios Papadimas, Spyridon Polykalas, Ioakeim Sapantzoglou, Aristotelis-Marios Koulakmanidis, Diamantis Athanasiou, Vasiliki Kanaka, Maria Kanaka, Kyriakos Bananis, Antonia Athanasiou, Aikaterini Athanasiou, Ioannis Papapanagiotou, Charalampos Tsimpoukelis, Maria Anastasia Daskalaki, Marianna Theodora, Nikolaos Thomakos, Panagiotis Antsaklis, Dimitrios Loutradis, Georgios Daskalakis

**Affiliations:** 1 1st Department of Obstetrics and Gynecology, ‘Alexandra’ General Hospital, National and Kapodistrian University of Athens, 80 Vasilissis Sofias Avenue, 11528 Athens, Greece; kimsap1990@hotmail.com (I.S.); aristoteliskoulak@gmail.com (A.-M.K.); vasilikikanaka@outlook.com (V.K.); maira.kanaka24@gmail.com (M.K.); tsimpoukelischa@gmail.com (C.T.); md181341@students.euc.ac.cy (M.A.D.); martheodr@gmail.com (M.T.); thomakir@hotmail.com (N.T.); gdaskalakis@yahoo.com (G.D.); 2 Laboratory of Forensic Medicine and Toxicology, School of Medicine, Aristotle University of Thessaloniki, 54124 Thessaloniki, Greece; fotischatzin@auth.gr (F.C.); loutradi@otenet.gr (D.L.); 3 Athens Medical School, National and Kapodistrian University of Athens, 15772 Athens, Greece; dr.georgepapadimas@gmail.com (G.P.); spyridonpolykalas@hotmail.com (S.P.); gpapamd@hotmail.com (I.P.); 4 IVF Athens Reproduction Center, 15123 Maroussi, Greece; diamathan16@gmail.com (D.A.); antoathan16@gmail.com (A.A.); diamathan17@gmail.com (A.A.); 5 King’s College Hospitals NHS Foundation Trust, London SE5 9RS, UK; kyriakos.bananis@nhs.net; 6 Fertility Institute-Assisted Reproduction Unit, Paster 15, 11528 Athens, Greece

**Keywords:** oxidative stress, telomeres, telomerase activity, granulosa cells, cumulus–oocyte complex, follicular fluid, oocyte quality, reproductive aging, mitochondrial dysfunction, DNA damage (8-OHdG), shelterin complex, SIRT1-FOXO-NRF2-AMPK axis, assisted reproductive technology, in vitro fertilization, ovarian reserve, biological ovarian aging, polycystic ovary syndrome, endometriosis

## Abstract

The reduction in oocyte competence and ovarian reserve coincides with reproductive ageing; nevertheless, the molecular mechanisms underlying this phenomenon remain poorly understood. Our testable mechanistic hypothesis is that the oxidative stress–telomere axis is a crucial regulatory mechanism controlling meiotic stability, mitochondrial resilience, and granulosa cell integrity. This notion posits that granulosa and cumulus cells have accelerated telomere attrition and impaired DNA-damage responses due to elevated amounts of reactive oxygen species, which also induce oxidative guanine lesions, inhibit telomerase function, and generate telomeric replication stress. This telomere-dependent vulnerability is anticipated to compromise developmental competence, disrupt meiotic spindle integrity, and diminish metabolic support to the oocyte, prior to observable declines in AMH or follicle count. Data from human IVF cohorts supports the model: Conditions such as PCOS, endometriosis, and POI have unique oxidative-telomeric profiles, whereas diminished telomere length in granulosa cells, reduced telomerase activity, and worse fertilisation, blastulation, and pregnancy outcomes are associated with increased follicular oxidative DNA damage. The findings suggest that oxidative DNA damage (8-OHdG), telomerase activity, and the structure of granulosa-cell telomeres may serve as preliminary indicators of preclinical ovarian ageing. This theory may be directly evaluated in forthcoming longitudinal studies and specific treatments related to telomerase regulation, mitochondrial medicines, or redox modulation. Consequently, the oxidative stress–telomere axis may represent a vital physiologic factor affecting reproductive lifespan and a prospective target for personalised ART techniques.

## 1. Introduction

### 1.1. The Role of Oxidative Stress in Ovarian Ageing

OS is becoming recognised as the molecular mechanism behind ovarian ageing and reproductive decline. Steroidogenesis, aerobic metabolism, and mitochondrial ATP generation continuously generate ROS in ovarian cells. An imbalance favouring oxidative species over antioxidant defences results in cellular and DNA damage [1]. This remains accurate despite the fact that proper amounts of reactive oxygen species are crucial for follicular signalling, meiotic spindle dynamics, and ovulation. The ovary is especially vulnerable to this imbalance because to its high metabolic demands, variable hormonal environment, and significant mitochondrial activity in both granulosa cells and oocytes [2].

Excessive ROS impair cellular function by inducing lipid peroxidation, protein oxidation, destabilising mitochondrial membranes, and causing direct DNA damage. Telomeres are particularly susceptible genomic areas because oxidative modifications preferentially affect their guanine-rich sequences [3]. Oxidative lesions, including 8-hydroxy-2-deoxyguanosine (8-OHdG), preferentially accumulate at telomeric DNA, obstructing the progression of replication forks, undermining telomere capping, and accelerating telomere shortening regardless of cell division [4]. Granulosa and cumulus cells, essential supporting cells that maintain follicular health and oocyte competence, undergo cellular senescence, p53-mediated apoptosis, and early activation of DNA damage response pathways as a consequence [5].

Human studies further emphasise the pathogenic significance of oxidative stress in the ovarian milieu. The translational effect of oxidative DNA damage on reproductive outcomes is shown by the correlation between increased 8-OHdG levels in granulosa cells and follicular fluid and a decrease in mature oocyte counts, poorer fertilisation rates, and reduced blastocyst formation in IVF cycles [6]. In pathological situations like endometriosis and PCOS, impaired mitochondrial function and chronic inflammatory signalling increase the amounts of ROS. This results in the early senescence of granulosa cells, accelerated telomere degradation, altered expression of telomere-protective proteins (including TRF1/TRF2), and the overexpression of pro-apoptotic markers. These mechanisms likely contribute to the infertility and diminished oocyte quality often reported in these circumstances [7].

Oxidative stress is significant since it impairs telomeric DNA and inhibits telomerase activity, hence complicating the maintenance of telomere integrity. These two processes enable oxidative stress to accelerate telomere attrition both directly and indirectly [8]. Antioxidant therapy, such as melatonin, mitochondrial-targeted antioxidants, and SIRT1 activation, have been effective in reducing oxidative damage, partly restoring telomere stability, and enhancing the cellular viability and metabolic function of ovarian tissue [9]. Lifestyle variables, including nutrition, exercise, and metabolic management, influence redox balance and telomere dynamics. This indicates that certain biological and behavioural modifications may mitigate reproductive ageing. OS is a crucial pathway connecting ovarian telomere failure, metabolic dysregulation, environmental exposures, and inflammatory signalling. Oocyte quality is diminished by oxidative stress, which impedes energy-dependent meiotic processes and hastens the onset of cellular senescence via cumulative mitochondrial and genomic damage, eventually leading to decreased reproductive capacity [10]. A comprehension of oxidative-stress mechanisms within the ovarian microenvironment provides a conceptual foundation for the development of diagnostics and treatment strategies focused on maintaining telomere integrity and enhancing IVF results, while also clarifying critical elements of reproductive ageing.

### 1.2. Vulnerabilities of the Ovarian Microenvironment: Granulosa Cells, Cumulus Cells, and Oocytes

The ovarian follicle is among the most metabolically active and physiologically responsive microenvironments in the body. It comprises a highly specialised assemblage of three cell types: granulosa cells, cumulus cells, and the oocyte [11]. These cells must collaborate for successful follicular growth, meiotic maturation, and early embryogenesis. This milieu is particularly susceptible to oxidative, metabolic, and inflammatory stress owing to the extended lifetime of oocytes, the significant proliferative requirements of somatic follicular cells, and the cyclical hormonal stimulation that promotes follicular recruitment and ovulation [6].

Granulosa cells (GCs) play a crucial role in orchestrating follicular development, providing metabolic support, synthesising oestradiol, and facilitating communication with the oocyte via paracrine signalling. Their incessant mitotic activity renders them very susceptible to oxidative DNA damage and telomere attrition that occurs during replication [12]. Oxidative stress overwhelms antioxidant defences, resulting in the oxidation of telomeric guanine residues, cessation of replication forks, and activation of checkpoint pathways. This induces GC senescence or apoptosis prematurely. Compromised GC function ultimately impairs fertilisation and embryo development by affecting mitochondrial transfer, cytoplasmic maturation, and the metabolic and hormonal support provided to the egg [13].

The cumulus–oocyte complex (COC) comprises CCs, a specialised subset of granulosa cells that closely encircle the oocyte and establish metabolic and signalling connections with the germ cell. These cells participate in cAMP signalling, essential for oocyte meiotic arrest and rapid resumption, and facilitate the transport of cholesterol, amino acids, and pyruvate to the oocyte [14]. Due to their proximity to the oocyte, cumulus cells may transmit oxidative damage, telomere attrition, or inflammatory stress, resulting in the direct delivery of reactive oxygen species, metabolic deficiency, and mitochondrial dysfunction to the ooplasm [15]. Patients with endometriosis exhibit that CC senescence, triggered by ROS-mediated endoplasmic reticulum stress, results in decreased ATP levels, impaired mitochondrial membrane potential, and a lower yield of mature oocytes. Antioxidant therapy mitigates these effects. These results suggest that CCs function as sensitive early markers of impaired oocyte support and as sentinel cells for oxidative damage [16].

Oocytes have a distinct biological vulnerability since they must maintain mitochondrial activity and genomic integrity throughout the extended meiotic halt. Oocytes, in contrast to somatic cells, primarily rely on pre-existing mitochondrial populations that cannot be supplied after birth and have little capacity for DNA repair. As a result, they are particularly vulnerable to accumulated oxidative and telomeric DNA damage. Spindle instability, kinetochore malfunction, mitochondrial insufficiency, and chromosomal segregation mistakes are molecular traits of ageing oocytes that are intimately associated with aneuploidy and infertility. Disrupted telomere homeostasis, either from oxidative erosion or diminished telomerase activity, jeopardises chromosomal stability and meiotic integrity.

## 2. Material and Methods

This narrative review sought to consolidate clinical, translational, and molecular findings regarding the oxidative stress–telomere axis in female reproductive ageing and assisted reproduction. A narrative review approach was chosen to enable a thorough assessment of innovative biological concepts and their significance in clinical practice, especially in a field marked by varied experimental methods, biomarker platforms, and clinical outcomes that hinder the strict uniformity necessary for systematic meta-analysis.

## 3. The Biology of Telomeres and Reproductive Physiology

Telomeres are crucial protective structures at the termini of chromosomes that maintain genomic stability during cellular division. They consist of repeating TTAGGG sequences and analogous shelterin proteins [17]. Their integrity inhibits growing genomic instability, inappropriate activation of DNA repair mechanisms, and chromosome-end fusion. Telomeres play a crucial role in reproductive physiology, since the ovarian follicle and germline represent distinct biological compartments where genomic integrity is essential for effective gametogenesis and embryonic development [18]. Germ cells, in contrast to somatic tissues, must preserve genetic integrity during decades of metabolic activity and cellular dormancy. Thus, the telomere dynamics of ovarian tissue are inherently different from those seen in other ageing systems.

Granulosa cells, essential for folliculogenesis, proliferate rapidly by mitosis during follicle development. These cells are more susceptible to cumulative shortening caused by telomere attrition that occurs with each mitotic event. In granulosa cells, the loss of telomere integrity induces apoptotic signalling, activates p53, and triggers DNA damage pathways, hence diminishing follicular functionality and the developmental potential of oocytes [19]. Cumulus cells maintain proximity to the oocyte via paracrine signalling and gap junctions, and need intact telomeres for cytoplasmic maturation, meiotic control, and nutrition transfer. The ovarian microenvironment is intricately interwoven, shown by the transfer of dysfunction from impaired telomeres in somatic ovarian cells to the egg [20].

The oocyte poses a distinct biological challenge. Oocytes in meiosis I that remain arrested for years or decades acquire oxidative and metabolic stress without the ability to divide, so making telomere preservation dependent on intrinsic quality rather than proliferative renewal [21]. Telomerase activity is elevated in early germ cells and follicular phases, contributing to telomere elongation. However, this activity diminishes as the ovaries age. Deficiencies in the meiotic spindle, improper chromosomal segregation, and aneuploidy conditions intricately associated with maternal age and diminished fertility occur more often when telomeres reach dangerously low lengths. Thus, ovarian reserve, oocyte competence, and reproductive lifetime are tightly linked to telomere biology [22].

An increasing volume of clinical and translational research indicates that telomere length and telomerase activity in ovarian cells correlate with a woman’s fertility potential. Investigations into the telomeres of granulosa cells have shown a correlation between longer telomeres, increased telomerase activity, and improved IVF outcomes, including elevated rates of oocyte maturation and fertilization [23]. In contrast, women with early ovarian insufficiency and low ovarian reserve have decreased telomerase activity, reinforcing the concept that telomere dysfunction accelerates reproductive ageing. However, the tissue-specific attributes of telomere control are highlighted by the finding that systemic markers, such as leukocyte telomere length, often have poor or inconsistent associations with reproductive outcomes. These data substantiate the notion that ovarian telomere biology serves as a superior metric for assessing reproductive ageing compared to peripheral indicators [24]. The concept that telomere preservation is crucial for maintaining the cellular integrity of the ovarian niche and safeguarding germline genomic stability is supported by current evidence. Telomeres and telomerase serve as molecular guardians that connect cellular ageing, metabolic stress, and reproductive viability. As research progresses, their incorporation into fertility assessment frameworks may provide a physiologically based alternative to conventional endocrine indicators, connecting clinical reproductive medicine with molecular ageing research [24].

### 3.1. The Potential of Oocytes and Telomerase Activity

Telomerase, a ribonucleoprotein enzyme consisting of the RNA template TERC and the catalytic subunit TERT, is essential for preserving chromosomal integrity and extending telomeres in the ovary. Ovarian tissue sustains telomerase expression, particularly in granulosa cells and early germ cells, unlike other somatic cells where telomerase is mostly dormant [25]. This selective preservation demonstrates the importance of maintaining genomic stability over the prolonged reproductive period and the fast proliferation of somatic follicular cells during each cycle of folliculogenesis. A recent research reveals that telomerase has activities beyond just elongating telomeres. It protects oocytes against oxidative stress, controls cellular senescence pathways, and modifies mitochondrial signalling [18].

Clinical studies suggest that telomerase activity is a more dynamic and clinically relevant indicator of ovarian function than the evaluation of static telomere length. Although there was no significant difference in telomere length across the groups, women who became pregnant in a crucial IVF study had markedly elevated telomerase activity in granulosa cells compared to non-pregnant patients [18,26]. It is essential to acknowledge that telomerase may provide predictive functions, since a one-unit increase in telomerase activity is associated with a five-fold increase in the probability of conception. The observations suggest that telomerase may work as an initial compensating mechanism inside the ovarian microenvironment, maintaining cellular function and telomere homeostasis before reaching a critical telomere length threshold [27].

Telomerase seems to influence essential embryological parameters beyond oocyte viability. Granulosa-cell telomerase activity is associated with better oocyte maturation, elevated fertilisation rates, and enhanced early embryonic development. This suggests that telomerase-mediated genomic preservation in the follicular compartment affects embryo viability [28]. These links likely indicate less oxidative DNA damage, sustained mitochondrial function, and continuous metabolic support for the developing oocyte from granulosa and cumulus cells. Research on reproductive disorders, particularly early ovarian insufficiency marked by diminished telomerase activity, underscores the essential role of telomerase in maintaining ovarian function [29].

Nonetheless, not all studies demonstrate analogous associations between telomerase activity and clinical outcomes, these discrepancies often arise from variations in tissue selection, used methodologies, and patient demographics. Measurements obtained from granulosa cells or follicular fluid more accurately reflect the local microenvironment; yet, leukocyte telomerase activity or telomere length may not consistently signify the biological age of the ovary. These results underscore the importance of selecting physiologically relevant tissue compartments when examining telomere-based reproductive markers [30].

The results indicate that telomerase is essential for sustaining oocyte competence by enhancing cellular viability in the ovarian niche, safeguarding the genome, and modulating redox balance. Ongoing research suggests that detecting telomerase in granulosa cells might enhance standard ovarian reserve markers, particularly for evaluating individuals with recurrent poor egg maturation, variable ovarian response, or unexplained infertility. Additional research is necessary to ascertain if pharmaceutical modulation of telomerase activity, dietary changes, or lifestyle adjustments might prolong ovarian longevity and improve reproductive outcomes.

### 3.2. Telomere Length as an Indicator of Ovarian Reserve and IVF Efficacy

Telomere length is essential for maintaining chromosomal integrity and cellular survival, prompting significant investigation into its potential as a biomarker for ovarian reserve and reproductive capability. Ovarian somatic cells with elongated telomeres theoretically have enhanced replication capabilities and improved DNA repair mechanisms [31]. Conversely, cells with abbreviated telomeres exhibit indications of senescence, diminished follicular quality, and an elevated chance of meiotic errors. This notion corresponds with essential reproductive biology: throughout the reproductive lifetime, the ovary experiences considerable metabolic stress, oxidative exposure, and mitotic activity, making telomeres more susceptible to degradation. Thus, telomere length has garnered considerable interest as a potential marker of biological ovarian ageing that surpasses conventional hormonal and ultrasonographic measures [32].

Data from human IVF cohorts suggest a relationship between ovarian cell telomere length and oocyte developmental competency. Investigations into granulosa and cumulus cell telomeres have shown that elongated telomeres correlate with increased rates of oocyte maturation, fertilisation, and embryonic development [33]. Recent evidence demonstrates a favourable correlation between granulosa-cell telomere length and both maturation and fertilisation rates. Its diagnostic accuracy in predicting oocyte quality is comparable to that of recognised ovarian markers. The correlation between telomere shortening and accelerated reproductive ageing is reinforced by evidence that women with early ovarian insufficiency and diminished ovarian reserve have granulosa-cell telomere shortening [34]. These results highlight tissue specificity as a crucial factor in telomere-based reproductive assessment, indicating that telomere length in follicular cells more accurately reflects the ovarian microenvironment than systemic assessments.

The efficacy of peripheral leukocyte telomere length as an indicator of ovarian age remains contentious. Leukocyte telomere length has a poor or insignificant link with fecundability, live birth rates, or miscarriage risk in women using ART or pursuing natural conception [35]. Leukocyte telomere dynamics have shown to be an inconsistent predictor of infertility or the need for assisted reproductive technologies in large population-based cohorts. These inequalities presumably stem from intrinsic biological distinctions between ovarian and haematopoietic tissues, including changes in oxidative exposures, DNA repair processes, and telomerase activity profiles [36]. The need to assess telomere biology in reproductive organs is highlighted by the finding that, whereas leukocyte telomere length may relate to systemic ageing and lifespan, its connection to ovarian function seems to be, at most, indirect.

The heterogeneity in reported findings is due to methodological differences and biological variability. Telomere length may be assessed by many methods, including fluorescence in situ hybridisation, quantitative PCR, and terminal restriction fragment analysis. Each has distinct strengths and shortcomings regarding repeatability and sensitivity [37]. Variations in patient phenotype, assay platform, and the examined cell population might alter findings and complicate the comparison of research. Furthermore, telomere length, as a static metric, inadequately reflects the dynamic balance between erosion and repair driven by telomerase activity. Thus, telomere length alone may not provide a thorough evaluation of ovarian biological age unless examined with telomerase activity and oxidative stress markers [38].

Notwithstanding these constraints, a growing corpus of research indicates that telomere length may function as a biomarker for oocyte quality and IVF outcomes, especially in granulosa and cumulus cells. Subsequent research connecting telomere length, telomerase activity, oxidative stress markers, and clinical outcomes may provide a more sophisticated and physiologically responsive assessment instrument for reproductive medicine. Standardised measurement techniques, rigorous validation across varied IVF populations, and integration with other molecular and metabolic markers of ovarian function are crucial for the therapeutic use of telomere measurements.

### 3.3. Senescence of Granulosa Cells, Telomeres, and Oxidative Stress

OS is the primary biochemical factor contributing to reproductive ageing, since it impairs telomeric DNA, inhibits telomere maintenance processes, and accelerates the ageing of GCs. Telomeres are particularly susceptible to oxidative alterations, such as the production of 8-hydroxy-2-deoxyguanosine (8-OHdG), owing to their high guanine content [39]. These defects impede telomeric replication and destabilise the protective shelterin complex, particularly TRF1 and TRF2. This results in the stalling of replication forks, the disintegration of t-loop structures, and the exposure of chromosomal ends. Experimental study demonstrates that oxidative telomeric damage mostly triggers γH2AX signalling rather than 53BP1 accumulation, suggesting a replication-stress phenotype rather than conventional double-strand breaks [8]. By facilitating chromatin remodelling into a restrictive state marked by elevated H3K9me3, these telomere-specific replication barriers eventually trigger cell-cycle arrest via p53–p21 activation. This results in reduced metabolic collaboration with the oocyte, accelerated depletion of granulosa cell proliferative ability, and progressive reduction in meiotic maturation competence, which is a key feature of reproductive decline in the ovary [40].

Human clinical data validate these mechanistic findings. The simultaneous measurement of telomere biology and oxidative stress markers in granulosa cells and FF highlighted the follicular microenvironment as a significant locus of oxidative stress in IVF patients. The results demonstrated that 8-OHdG levels were significantly increased in follicular fluid compared to granulosa cells [18]. Significantly, in both GCs and FF, GC 8-OHdG demonstrated an adverse relationship with telomerase activity, suggesting that oxidative stress not only damages telomeric DNA but also impedes intrinsic telomere repair processes. Increased oxidative DNA damage was directly associated with reduced maturation (MII) rates, delayed blastocyst development, fewer fertilisations, and a decreased number of recovered oocytes [41]. These results illustrate a clinically relevant sequence wherein oxidative stress alters the telomere biology of ovarian somatic cells, resulting in quantifiable decreases in oocyte competence and early embryonic performance, prior to any detectable drop in follicle number.

Pathological reproductive phenotypes clarify the OS–telomere relationship. Granulosa and cumulus cells in PCOS demonstrate shorter telomeres, modified expression of telomere-protective proteins (TRF1/TRF2), and increased apoptotic signalling pathways due to oxidative stress-induced telomere erosion and premature cellular senescence [42]. The identified mitochondrial dysfunction and increased ROS production in PCOS granulosa cells indicate that prolonged oxidative signalling plays a role in abnormal folliculogenesis and reduced oocyte quality, transcending simple endocrine and metabolic factors [43]. Similarly, chronic inflammation and redox stress in endometriosis induce ageing in granulosa cells via endoplasmic reticulum stress, resulting in ATP depletion, mitochondrial membrane potential collapse, and a reduction in oocyte quantity [16]. Pharmacologic rescue tests demonstrate that melatonin alleviates endoplasmic reticulum stress, telomere dysfunction associated with oxidative stress, and mitochondrial and reproductive metrics in both living and nonliving cells. This suggests that certain redox therapies may mitigate telomere erosion in this context [44].

Oxidative stress linked to metabolic lifestyle impacts telomere integrity. Women exhibiting elevated follicular fluid glucose levels and superior metabolic characteristics have longer GC telomeres and significantly greater pregnancy rates. This suggests that telomere dynamics and ensuing reproductive capacity are affected by local nutritional conditions and overall metabolic stability [45]. The data suggest a bidirectional paradigm whereby telomere stability, oxidative status, and mitochondrial efficiency interact to influence follicular function and oocyte competence. This model may explain the differences in IVF results across patients, including those with comparable ovarian reserve and age. Metabolic modulation and redox optimisation may be effective techniques to preserve telomere structure in ovarian somatic cells and improve clinical reproductive outcomes [30].

Oxidative stress increases cellular vulnerability by directly inhibiting telomerase activity, the enzyme that safeguards telomere length, while also causing structural damage to telomeres. Studies on IVF cohorts reveal that, irrespective of differences in average telomere length among groups, heightened granulosa-cell telomerase activity independently predicts improved oocyte yield, higher blastocyst transfer rates, and roughly a five-fold increase in the probability of clinical pregnancy [19]. This highlights that, unlike static telomere structure, telomerase activity functions as a dynamic measure of resilience, reflecting the ability of ovarian cells to withstand oxidative and replicative stress. Supplementary results suggest that the telomere length of granulosa cells is positively associated with fertilisation and oocyte maturation rates, providing a notable diagnostic discriminative ability (AUC ~0.72 for forecasting oocyte maturity) [34]. All these findings indicate a unified biological model: oxidative stress induces telomerase suppression and expedited telomeric degradation, resulting in granulosa cell senescence, mitochondrial malfunction, and diminished oocyte developmental competence. This cascade results in diminished probabilities of fertilisation, protracted embryo development, and reduced likelihood of a successful pregnancy [46].

## 4. Oxidative Stress in Female Reproduction

We propose a comprehensive mechanistic framework for the oxidative stress–telomere axis, conceptualising the system as consisting of three interconnected feedback loops operating inside the physiologically hypoxic ovarian follicle. Initially, early telomere failure is induced by an increased production of ROS by mitochondria. This occurs because to steroidogenesis, an elevated demand for ATP, and a hypoxic environment inside the follicle. This results in the cessation of the replication fork and the oxidation of guanine at telomeric repeats. Secondly, oxidative damage to telomeres accelerates the senescence of granulosa cells and impedes oocyte support by inhibiting telomerase activity, disrupting shelterin-mediated capping, and activating DNA damage signalling pathways. Third, telomere failure contributes to the accumulation of ROS by impairing mitochondrial DNA, resulting in decreased TERT levels and diminished membrane potential. Oxidative damage, telomeric instability, and mitochondrial dysfunction mutually worsen one another over time, creating a self-perpetuating cycle via these interrelated mechanisms. This model clarifies the process via which telomere driven reproductive ageing might advance prior to detectable reductions in endocrine markers or ovarian reserve. This mechanistic characterisation establishes a conceptual basis for the oxidative stress telomere axis, underscoring its potential significance as an upstream regulatory network influencing follicular lifespan and oocyte competence.

### 4.1. Conceptual Framework and Sources of ROS in the Ovary

The generation of ROS and antioxidant defences is delicately imbalanced, leading to oxidative stress, a significant biochemical contributor to female reproductive ageing and the decline of oocyte quality [47]. Folliculogenesis, steroidogenesis, and meiotic maturation are activities inherently linked to heightened metabolic turnover, sustained mitochondrial activity, and dynamic endocrine signalling, making this equilibrium especially delicate in the ovary. The ovarian follicle, in contrast to numerous somatic tissues, operates within a microenvironment where ROS act as physiological signalling molecules crucial for normal reproductive functions, such as luteinisation, steroid hormone production, and follicular rupture during ovulation, while also serving as metabolic byproducts [48]. When the formation of ROS surpasses the detoxifying capacity, these free radicals trigger metabolic events that harm proteins, lipids, and DNA, especially the telomeres of granulosa and cumulus cells, which are particularly vulnerable owing to their guanine-rich composition [49].

Mitochondrial oxidative phosphorylation is a primary mechanism by which the ovaries generate ROS. Superoxide is generated when electrons escape from complexes I and III, particularly during periods of high ATP demand, such as during meiotic spindle formation or follicle development [50]. Steroidogenic enzymes, particularly cytochrome P450 isoforms involved in oestrogen production, generate hydrogen peroxide as a reaction intermediate. The follicular microenvironment experiences accumulated oxidative damage from decades of repeated inflammatory oxidative exposure, even though it is crucial for ovulation [51].

Systemic metabolic conditions also influence ovarian oxidative balance. Insulin resistance, obesity, and dyslipidaemia increase ROS levels by imposing additional stress on mitochondria, disrupting β-oxidation, and inducing chronic low-grade inflammation [52]. These reactive oxygen species then permeate the ovarian stroma and follicular fluid. Environmental variables like pollution, tobacco smoke, endocrine disruptors, and chemotherapeutic drugs increase oxidative stress. The egg lacks the proliferative turnover and DNA repair redundancy typical of somatic cells, making it more susceptible to oxidative damage buildup owing to its prolonged meiotic halt from foetal development to reproductive maturity [53].

Minor alterations in oxidative excess can accelerate telomere degradation, disrupt chromosomal cohesion, and induce granulosa-cell senescence within this reproductive environment characterised by persistent mitochondrial stress, cyclical inflammatory episodes, hormone-mediated metabolic fluctuations, and environmental and systemic oxidative influences [15]. In this scenario, oxidative stress functions as a significant factor influencing IVF results and reproductive longevity, rather than just a passive association with ageing. Reproductive ageing is more directly linked to biochemical burden than to chronological age, since the ovary may be considered a tissue that ages not just over time but also due to accumulated oxidative stress in relation to metabolic activity.

Alongside the conventional ROS pathways, the L-arginine–NO system serves as an additional regulatory axis that influences the redox equilibrium of the ovaries and the viability of the follicles. Nitric oxide is implicated in mitochondrial signalling, regulating the vascular milieu of the follicles, and facilitating meiotic maturation. Nonetheless, excessive synthesis of nitric oxide or peroxynitrite might intensify oxidative stress and diminish oocyte quality. Reactive nitrogen species derived from nitric oxide influence antioxidant networks, mitochondrial membranes, and telomeric DNA. This indicates that the L-arginine–NO system may influence telomere integrity either directly or indirectly. Recent evaluations suggest that impaired reproductive efficacy and disrupted folliculogenesis arise from aberrant L-arginine metabolism, dysregulation of NO production, and altered activity of NOS isoforms [54]. Thus, including nitric oxide biology into oxidative stress models enhances the knowledge of the molecular pathways influencing granulosa cell function and oocyte survival.

### 4.2. Translational Readouts and the Redox Biology of Follicular Fluid

FF is the biochemical environment that surrounds the developing oocyte. It is where metabolic substrates, signalling molecules, detoxification systems, and nutrient exchange all happen. So, it shows the integrated oxidative status of the follicular niche more accurately than systemic blood biomarkers [55]. FF keeps antioxidants that are released when oxidative stressors and ROS are made by granulosa and cumulus cells. FF is not just a passive reservoir, it is a functional oxidative micro-ecosystem where the redox balance directly affects oocyte competence [56].

A growing body of IVF-based research shows that FF oxidative markers are strongly linked to important reproductive outcomes. It is interesting that FF cells always have more 8-hydroxy-2-deoxyguanosine (8-OHdG), a very specific biomarker of oxidative DNA damage, than granulosa cells do [57]. This pattern suggests that the extracellular environment may act as an oxidative buffer, absorbing damage and offering some protection to the oocyte, but ultimately reaching a saturation point that harms nearby cells. Research indicates a direct biochemical correlation between oxidative damage and suboptimal reproductive performance, as elevated levels of FF 8-OHdG are associated with reduced blastocyst formation, diminished MII generation, decreased total oocyte yield, and compromised fertilisation rates. Oxidative damage in FF has been demonstrated to exert independent effects that transcend ovarian reserve and chronological age, thereby establishing OS as a distinct biological axis regulating oocyte quality [58].

Excessive FF ROS interferes with the oocyte’s spindle formation, meiotic chromosomal segregation, and mitochondrial membrane potential at a mechanistic level. These disruptions hinder ATP production and destabilise microtubules necessary for accurate meiotic division, both of which are critical biological processes for successful fertilisation and embryonic development [56]. Additionally, even when nuclear maturation proceeds normally, oxidative stress in FF compromises oocyte cytoplasmic maturation by preventing pyruvate transfer and affecting the metabolic crosstalk between cumulus cells and the oocyte. This uncoupling between cytoplasmic and nuclear readiness is a sign of suboptimal oocyte competence, and it is becoming more widely accepted in IVF labs [59]. FF is also full of molecular antioxidants like melatonin, vitamin E, and glutathione, as well as antioxidant enzymes like catalase, glutathione peroxidase, and superoxide dismutase. Follicles with a high level of competence seem to be different from those that are likely to fail to develop because of how many and how active these defence mechanisms are. In diseases like PCOS and endometriosis, the FF antioxidant capacity is often lower and the oxidative load is higher. This imbalance diminishes the redox safety margin within the follicular microenvironment [60]. These findings align with translational research indicating that antioxidant supplementation, especially melatonin, enhances mitochondrial function, reduces oxidative DNA damage, and elevates oocyte quality in both human and animal models.

### 4.3. Interplay Between Mitochondria and Telomeres in Cumulus and Granulosa Cells

Mitochondria are crucial for controlling ovarian function since they produce ATP, essential for the energy-demanding processes of egg maturation, spindle formation, chromosomal segregation, and meiotic development [61]. Throughout folliculogenesis and steroidogenesis, GCs continuously sustain oxidative metabolism. Each mature egg has hundreds of thousands of mitochondria, resulting in an excessive mitochondrial burden inside the ovary. The ovarian microenvironment is very vulnerable to redox fluctuations due to elevated metabolic activity. Mitochondria serve as the primary producer of ROS and are the principal targets of oxidative damage. The interplay between ROS and mitochondrial activity creates a self-reinforcing cycle. When mitochondria malfunction, they produce excessive ROS, which exacerbate the condition of respiratory enzymes, mitochondrial membranes, and DNA, so impairing reproductive ability [61].

Telomerase, the enzyme responsible for elongating and repairing telomeres, has two crucial protective functions in ovarian tissue. Telomerase reverse transcriptase not only safeguards telomeric ends but also translocates to mitochondria, where it diminishes ROS generation, preserves mitochondrial DNA, and maintains membrane potential stability [62]. Consequently, the suppression or shortage of telomerase under oxidative stress results in a twofold disadvantage: diminished mitochondrial robustness and insufficient telomere preservation. The dynamic capabilities of telomere repair and mitochondrial protection may possess greater biological relevance than telomere length alone, as evidenced by human IVF studies showing that granulosa-cell telomerase activity is more closely associated with oocyte maturation and pregnancy outcomes than static telomere length [18]. This aligns with research suggesting that, despite comparable ovarian reserve and chronological age, individuals exhibiting heightened GC telomerase activity have improved oocyte yield and much greater chances of achieving clinical pregnancy. Mitochondrial-telomeric crosstalk elucidates the processes underpinning reproductive abnormalities in metabolic and inflammatory diseases [63]. Despite the presence of many follicles in the ovaries, PCOS leads to their premature failure due to issues with shelterin (alterations in TRF1/TRF2 expression), mitochondrial dysfunction, excessive ROS generation, and aberrant redox signalling. Melatonin supplementation mitigates ATP depletion, depolarised mitochondrial membranes, and accelerated telomere attrition caused by ROS-mediated endoplasmic reticulum stress and mitochondrial dysfunction in granulosa cells linked to endometriosis, highlighting the therapeutic importance of maintaining mitochondrial integrity for telomere stability [64].

### 4.4. Disease Models Linking Oxidative Stress, Telomere Dysfunction, and Reproductive Decline

Reproductive illnesses such as PCOS, endometriosis, and POI are clinically significant human models that illustrate the interplay between oxidative stress and telomere biology in undermining ovarian function. Notwithstanding variations in aetiology and clinical manifestation, a shared molecular theme emerges: persistent oxidative stress precipitates telomeric erosion, granulosa cell senescence, mitochondrial dysfunction, and eventually impedes egg maturation and fertility [65]. These pathophysiological pathways accelerate the physiological ageing process, suggesting that disease-related telomere dynamics may mimic and often hasten the molecular features of ovarian ageing. PCOS is characterised by a chronic hyperandrogenic and metabolic inflammatory state that intensifies oxidative stress in the ovaries. Granulosa cells from individuals with PCOS have increased reactive oxygen species, impaired mitochondrial activity, and reduced antioxidant defences. Recent experimental data indicates that telomere-regulating proteins are impaired in PCOS [20]. Cumulus cells have elevated TRF1 levels, modified TRF2 expression, and increased BAX, an apoptotic marker. Moreover, telomeres are much shorter than those in the control group. These alterations indicate that cellular ageing is accelerating, despite the increase in follicle count, which is perplexing. This discordance underscores a crucial insight: follicular richness does not correspond with cellular vitality or telomere integrity [66]. Furthermore, mitochondrial-telomere damage in PCOS may impede egg maturation and fertilisation efficacy, despite a seemingly adequate ovarian reserve. This shift in perspective elucidates why individuals with PCOS continue to have difficulties in conceiving while using ART, and it implies that telomere-supportive interventions may synergise well with metabolic treatment in this context [39].

Endometriosis demonstrates a distinct mechanism of ovarian oxidative injury: an inflammatory pelvic milieu and ectopic endometrial tissue generate sustained ROS exposure that infiltrates the follicular niche. Granulosa cells from patients with endometriosis have elevated senescence-associated β-galactosidase activity, elevation of endoplasmic reticulum stress indicators, deterioration of mitochondrial membrane potential, and decreased ATP levels [67]. This ageing phenotype is linked to telomeric degradation and reduced oocyte competence. Mechanistic studies reveal that the modulation of oxidative-endoplasmic reticulum stress signalling reverses granulosa cell senescence, while melatonin supplementation an antioxidant aimed at mitochondria normalizes telomere stress indicators and restores energy metabolism. These findings endorse a treatment strategy: reinstating mitochondrial and telomeric integrity may be essential for improving IVF outcomes in endometriosis, especially in individuals with repeated suboptimal responses or reduced egg maturity rates [68]. POI provides the most persuasive evidence for the role of telomeres in ovarian failure. Multiple observational cohorts have shown that women with POI consistently have reduced telomere length and decreased telomerase activity in leukocytes and granulosa cells. Telomere attrition precedes follicular depletion, rendering POI an expedited model of ovarian ageing, whereby oxidative stress, inadequate DNA repair, and diminished telomerase converge to precipitate an earlier-than-anticipated collapse of follicular reserve [30]. This substantiates the notion that telomere length may serve as a valuable biomarker for forecasting ovarian longevity and the onset of reproductive decline, in conjunction with AMH and antral follicle count. Nonetheless, the inconsistency among research necessitates uniform assessment procedures and prospective validation before therapeutic incorporation.

### 4.5. Principal Antioxidant Signalling Mechanisms That Preserve the Integrity of Telomeres in Ovarian Cells

The ability of granulosa cells, cumulus cells, and oocytes to neutralise ROS is a crucial factor in reproductive success, since the ovarian microenvironment is particularly susceptible to oxidative damage. To protect telomeres, a well-coordinated network of intracellular antioxidant pathways is essential. Telomeres are the intersection of oxidative stress and DNA damage [69]. The SIRT1-FOXO3-NRF2-AMPK axis is the most significant among them. It is a regulatory circuit that integrates mitochondrial health, redox state, metabolic signals, and DNA repair mechanisms. Understanding the functioning of these pathways is crucial, as they are consistently disrupted in instances of reproductive ageing, PCOS, endometriosis, POI, and suboptimal IVF response. Recent studies indicate that altering these pathways may enhance oocyte developmental competence, prevent granulosa cell senescence, and restore telomere stability [70].

NAD^+^-dependent deacetylation activates SIRT1 (Sirtuin-1), a principal regulator of mitochondrial function and longevity. SIRT1 regulates chromosomal integrity, enhances telomerase activity, and mitigates the accumulation of oxidative damage at telomeres in ovarian cells [71]. SIRT1 maintains telomere length and repairs oxidative guanine adducts via deacetylating and activating TERT. Simultaneously, it facilitates the formation of new mitochondria, elevates the levels of superoxide dismutase (SOD2), and inhibits pathways associated with ageing, including NF-κB inflammatory cascades and p53/p21 [72]. Studies have associated diminished SIRT1 expression with a decreased ovarian reserve, suboptimal IVF results, and impaired oocyte maturation. In contrast, pharmacological or dietary SIRT1 activators, such as nicotinamide riboside and resveratrol, have shown efficacy in restoring ovarian redox capability and telomere dynamics in experimental animals. SIRT1 is the primary mechanism by which the ovary protects itself against oxidative telomere damage [73].

The FOXO (Forkhead box O) transcription factors, particularly FOXO3a, collaborate with SIRT1 to regulate oocyte dormancy in primordial follicles and safeguard cells against oxidative stress. FOXO3 enhances the expression of DNA repair genes, telomere-protective mechanisms, and antioxidant enzymes such as SOD, catalase, and glutathione peroxidase (GPx) [74]. Its activation extends ovarian lifetime by preventing premature oocyte activation and depletion, while maintaining mitochondrial membrane potential. The critical role of FOXO3 in reproductive lifespan is highlighted by the accelerated follicular depletion and early ovarian senescence shown in FOXO3 knockout animals. FOXO3 interacts with SIRT1 [75]. The deacetylation induced by SIRT1 enhances the transcriptional activity of FOXO, establishing a robust oxidative defence mechanism that directly diminishes telomeric reactive oxygen species and preserves meiosis. NRF2 (Nuclear Factor Erythroid 2-Related Factor 2) serves as the principal transcriptional regulator of the body’s antioxidant responses. Upon translocation to the nucleus in reaction to oxidative stress, NRF2 activates detoxifying and redox-buffering enzymes, including heme oxygenase-1, glutathione-synthesizing enzymes, and NAD(P)H-dependent oxidoreductases. Activation of NRF2 in ovarian tissue prevents telomere shortening, sustains mitochondrial metabolism, and inhibits the accumulation of reactive oxygen species in granulosa cells. Research indicates that NRF2-targeted treatments such as melatonin, quercetin, and sulforaphane may enhance the integrity of ovarian telomeres and accelerate oocyte maturation in models of oxidative damage. Conversely, the loss of NRF2 accelerates follicle depletion, chromosomal mis-segregation, and oocyte apoptosis. Thus, the NRF2-telomere axis becomes a crucial target for preserving the genetic integrity of the ovaries [76].

AMPK (AMP-activated protein kinase), the primary indicator of a cell’s energy state, regulates metabolic balance and resilience to oxidative stress. Activation of AMPK enhances TERT expression and telomerase activity, reduces ROS generation by shifting metabolism towards oxidative processes, and accelerates mitochondrial turnover via mitophagy [77]. AMPK enhances telomere maintenance and mitochondrial repair via direct interaction with the SIRT1-PGC-1α signalling module. Impaired AMPK signalling in models of ovarian ageing and PCOS is associated with reduced oocyte competence, telomere degradation, and mitochondrial dysfunction. AMPK agonists, conversely, facilitate the rejuvenation of ovarian metabolism and preserve telomeres. Examples include metformin, calorie restriction mimetics, and exercise-mimetic compounds [78]. These pathways provide an integrated cellular network by integrating telomere maintenance and mitochondrial integrity. A prospective therapeutic approach to maintain reproductive lifespan, enhance follicular resilience, and improve IVF results entails targeting the SIRT1-FOXO0-NRF2-AMPK axis, considering the growing intersection of ovarian ageing and infertility with metabolic, inflammatory, and oxidative stress problems. The translational potential of treatments that modulate pathways, such as metabolic therapies, innovative senotherapeutics, and nutraceutical antioxidants, indicates a paradigm shift towards reproductive medicine driven by redox and telomere considerations [28].

### 4.6. Implications for IVF Clinical Practice and Innovative Biomarkers and Therapies

The incorporation of telomere biology and oxidative stress signalling into reproductive medicine signifies a significant transition from conventional evaluations of ovarian reserve to molecular indicators of oocyte quality, cellular resilience, and biological ovarian age. AMH, AFC, and chronological age inadequately represent the oxidative-telomeric stress load that progressively accumulates in ovarian tissue over time and under various pathological circumstances, despite their significance as clinical indicators [79]. The telomere-oxidative stress axis provides new prognostic tools and treatment options in IVF settings, where minor changes in cellular physiology may significantly affect fertilisation potential, embryo morphokinetics, and blastocyst viability. Evidence is mounting that ovarian ageing involves a qualitative decline in telomeric integrity, mitochondrial function, and genomic stability domains significantly influenced by oxidative metabolism rather than only a numeric decrease in follicle count [80].

Granulosa-cell telomerase activity is a promising biomarker. Research demonstrates that oocyte maturation rates, blastocyst development, and pregnancy outcomes are more closely associated with increased telomerase activity than with telomere length alone. This discovery highlights that dynamic telomere maintenance capability, rather than static telomere length assessment, functions as a more physiologically relevant indication of fertility [81]. Oxidative DNA damage is a direct predictor of IVF success, evidenced by significant negative correlations between oxidative DNA damage markers, such as 8-OHdG in follicular fluid, and metrics including retrieved oocyte count, mature (MII) oocyte yield, fertilisation outcomes, and blastocyst development. These biomarkers signify a more holistic assessment framework that amalgamates molecular indicators of oxidative resilience and cellular ageing with established ovarian reserve markers [82].

This molecular framework is now exhibiting translational medicinal relevance. Reproductive endocrinology has historically discussed antioxidant techniques, and they are presently improving by providing greater specificity on their mechanisms of action. In endometriosis models, melatonin, a powerful mitochondrial antioxidant, has shown the capacity to reverse granulosa cell senescence caused by ER stress, restore ATP production, and maintain telomere stability, therefore improving oocyte maturation [20]. Metabolic modulators like metformin improve mitochondrial efficiency and activate AMPK, possibly decreasing ROS levels and thereby safeguarding telomere integrity in PCOS. Enhancing one’s lifestyle via caloric reduction, a diet rich in antioxidants, regular exercise, and metabolic regulation may contribute to improved reproductive results and the postponement of ovarian telomere attrition [83]. Although broad antioxidant supplementation remains contentious due to its inconsistent efficacy, precision antioxidant treatment that specifically targets the SIRT1-FOXO-NRF2-AMPK axis and mitochondrial redox homeostasis is very promising.

Telomere-targeted medicines, including sirtuin agonists, small-molecule telomerase activators, senolytic or senomorphic drugs, and chemicals that augment DNA repair processes, may be used in future IVF operations [56]. Furthermore, in elderly or metabolically compromised individuals, pre-treatment ovarian “prehabilitation” protocols incorporating metabolic correction, mitochondrial enhancement, oxidative stress mitigation, and anti-inflammatory interventions prior to stimulation may enhance responsiveness and embryo quality. The notion of molecular ovarian age is becoming increasingly prominent, and the incorporation of telomere assessments with multi-omics methodologies (such as follicular-fluid secretomics, mitochondrial proteomics, redox metabolomics, and single-cell ovarian transcriptomics) may eventually enhance personalised controlled ovarian stimulation protocols, embryo selection techniques, and fertility preservation guidelines [28]. As the discipline advances, it must address the challenges of standardising assessments, establishing clinically relevant thresholds, and determining the optimal timeframe for effective intervention. Nevertheless, the growing data suggests that mitochondrial health, oxidative stress levels, and telomere function are concrete determinants affecting reproductive capacity rather than simply theoretical concepts. These molecular findings may enable the shift of IVF from a hormonally-driven approach to one centred on cellular rejuvenation and genetic safeguarding in clinical practice. This would provide more accurate predictions and customised therapies that improve reproductive lifespan and success rates [84].

At present, a restricted selection of prospective therapies aimed at the oxidative stress–telomere axis combines substantial molecular knowledge with nascent clinical data. In reproductive models, especially concerning endometriosis and those with suboptimal responses, melatonin-based antioxidant protocols provide the most consistent results. Melatonin improves the mitochondrial activity of granulosa cells, alleviates senescence caused by endoplasmic reticulum stress, and partly restores oocyte maturation and embryonic development. While precise telomere-centric outcomes remain uncommon, metabolic treatments such as metformin, which activate AMPK and improve insulin sensitivity, indirectly reduce mitochondrial ROS production in PCOS and may also subsequently safeguard telomere integrity. The predominant and safest methods to alter the redox load in the body and ovaries remain lifestyle modifications such as improved nutrition, weight reduction, physical exercise, and enough sleep. Conversely, direct telomerase-activating drugs, sirtuin agonists, potent antioxidants aimed at mitochondria, and mitophagy modulators remain in the first phases of investigation. We must evaluate the potential advantages of these medicines against the genuine risks of increasing cancer susceptibility, disrupting normal ageing, or causing unforeseen effects on mitochondria and epigenetics in the germline. Significant challenges in translating this research into practical application include insufficient data on dose-response and timing in IVF cycles, ambiguity regarding the optimal timing for treatment administration (prior to or during stimulation), and ethical and legal concerns associated with manipulating telomere and mitochondrial pathways in human gametes and embryos. Currently, ovary-proximal antioxidant and metabolic techniques seem to be the most promising for clinical use, whereas telomerase-targeted and intense mitochondrial treatments must remain subject to rigorous research procedures until their long-term safety is established.

## 5. Evidence from Human Studies

### 5.1. Telomere Dynamics and Chronological vs. Biological Ovarian Aging

Human reproductive ageing is widely acknowledged as a physiologically diverse process, where the deterioration of ovarian function and oocyte competence cannot be adequately represented by chronological age alone. Telomere biology, which indicates the accumulation of oxidative stress, the efficacy of DNA repair mechanisms, and cellular robustness, has emerged as a significant molecular marker of biological reproductive ageing [58]. Numerous human studies show that telomere attrition may act as a biological clock that more precisely reflects the depletion and decline of ovarian reserve compared to chronological age. A research investigating women who conceived naturally at late maternal ages (43–48 years) revealed a major clinical observation: their leukocyte telomere length (LTL) was much longer than that of age-matched women who could not conceive beyond age 41 [85]. The results show that inherent ovarian resilience is reflected by telomeric integrity, which may correlate with meiosis fidelity, extended oocyte genomic stability, and a decreased risk of age-related aneuploidy [86].

However, telomere biology cannot forecast reproductive results universally across all age demographics or clinical scenarios. In a large prospective cohort of younger women pursuing spontaneous conception, preconception leukocyte telomere length showed no association with time to pregnancy, pregnancy loss, or the likelihood of live delivery. The results suggest that telomere-based biomarkers may have clinical relevance only as reproductive ageing progresses or in women with subclinical ovarian loss, and that leukocyte telomere length may be ineffective in prediction when ovarian reserve is still strong [24]. This differentiation is further validated by extensive population-based reproductive epidemiology data. The Norwegian Mother, Father, and Child Cohort Study found no correlation between leukocyte telomere length and women’s fertility. It is noteworthy that males with longer telomeres had a greater propensity for seeking assisted reproduction, perhaps due to selected reproductive behaviours or paternal age effects rather than inherent fertility potential [87]. These results underscore a critical methodological and biological consideration: ovarian tissue, affected by particular metabolic and oxidative stresses, does not uniformly exhibit telomere control comparable to that of peripheral blood.

Current research shows that telomere degradation may achieve biological and clinical importance sooner in the reproductive process than previously anticipated. Observational studies demonstrate that granulosa cell telomere shortening and decreased telomerase activity begin in the mid-30s, many years before noticeable reductions in AMH or AFC occur. This aligns with research suggesting that telomere-dependent mechanisms, including mitochondrial malfunction, replication stress, and impaired DNA repair, start to affect oocyte competence prior to the noticeable depletion of follicles. Granulosa-cell telomerase activity seemingly decreases before telomere length stabilises, indicating accumulating oxidative damage and reduced cellular resilience. Despite the constancy of endocrine indicators, our findings indicate that telomerase activity may serve as an early indicator of preclinical ovarian ageing, capable of identifying women whose follicles are ageing at an accelerated rate. Telomerase based evaluations may potentially improve AMH and AFC by identifying ovarian vulnerability at a timeframe when mitochondrial, redox, and genetic therapies might still be effective; nevertheless, further prospective trials are required.

### 5.2. In Patients Undergoing In Vitro Fertilisation, Telomere Length and Telomerase Activity

The dynamics of telomeres in ovarian somatic cells particularly granulosa and cumulus cells, crucial for oocyte maturation and mitochondrial support have been directly assessed in assisted reproduction [88]. Evidence suggests that telomere length and telomerase activity are mechanistically significant to reproductive competence, however their prognostic value for IVF results varies according on the measured parameter and the clinical endpoint assessed. A groundbreaking research demonstrated that women who became pregnant after IVF had markedly increased telomerase activity in their luteinized granulosa cells relative to those who did not achieve conception [89]. TA significantly surpassed TL in predicting clinical pregnancy, exhibiting a superior AUC (0.674 compared to 0.576) and approximately quintupled pregnancy probabilities for each unit increase in TA [89]. Age, baseline FSH, and oestradiol levels failed to predict the outcomes, indicating that in IVF contexts, cellular repair capacity and telomere maintenance may be more clinically relevant than telomere length.

Subsequent prospective findings validate the functional significance of telomere biology at the follicular level. In a cohort of 240 IVF patients, granulosa-cell telomere length shown a favourable connection with the fertilisation rate (r = 0.408, *p* < 0.001) and the oocyte maturation rate (r = 0.386, *p* < 0.001), irrespective of conventional ovarian reserve metrics [81]. Telomeric length did not correspond with blastocyst formation or embryo quality, indicating that telomeric integrity mainly affects meiotic competence and cytoplasmic maturation, rather than later phases of embryonic development. The interplay between telomere control and oxidative stress clarifies this link [90]. A clinical investigation examining granulosa cells and follicular fluid from 102 IVF patients revealed that elevated levels of the oxidative DNA damage marker 8-OHdG correlated with a reduced number of recovered oocytes, mature oocytes, fertilisations, and blastocysts. Telomerase activity was reduced, and oxidative stress demonstrated an unfavourable relationship with telomerase activity [18]. This supports the idea that folliculogenesis may be considerably hindered by ROS-induced telomere disruption rather than just shortening. Supplementary clinical data substantiates these conclusions. Extended granulosa-cell telomeres were associated with increased follicular fluid glucose levels in IVF patients, indicating a superior metabolic environment and mitochondrial assistance. Moreover, women with prolonged telomere length had markedly elevated pregnancy rates (88% vs. to 38%) [45]. These findings support the idea that telomere stability, redox conditions, and bioenergetic state are interrelated.

Systemic telomere length may not precisely reflect the dynamic regulation of telomeres within the ovarian niche, as studies measuring telomeres in peripheral leukocytes show inconsistent correlations between telomere length, ovarian response, and embryo euploidy [91]. Granulosa cells are a more relevant tissue for evaluating reproductive telomeres, since they experience considerable mitotic and metabolic stress during gonadotropin stimulation, in contrast to leukocytes. Human studies suggest that oxidative-stress indicators and telomerase activity together have stronger relationships with IVF results than telomere length by alone [92]. Their main predictive importance is in detecting physiologically accelerated ovarian ageing, reduced fertilisation potential, and impaired oocyte maturation. Telomere-related biomarkers may function as supplementary instruments to AMH and AFC, especially for women with metabolic infertility, accelerated reproductive ageing, unexplained low fertilisation rates, or recurrent oocyte maturation failure [93]. The IVF cohorts together demonstrate that GC telomerase activity and telomere integrity surpass systemic indicators in predicting oocyte competence and early fertilisation outcomes (Table 1).

Due to the ovary being a separate metabolic and endocrine compartment with its own oxidative exposures, the regulation of ovarian telomeres is very different from the regulation of systemic telomeres. Cumulus and granulosa cells undergo significant mitochondrial turnover, steroidogenic activity, and multiple cycles of mitotic expansion, leading to a continuous redox load that leukocytes are unable to counterbalance. Additionally, oocytes exhibit a telomere–mitochondria interaction that is fundamentally distinct from that in somatic tissues, as they rely on a stable mitochondrial population with limited DNA-repair capabilities and remain meiotically arrested for decades. These characteristics explain why the local oxidative environment of individual follicles is not captured by leukocyte telomere length, which mainly reflects systemic oxidative and inflammatory ageing. Although no systemic biomarker fully reflects ovarian oxidative load, circulating 8-OHdG, malondialdehyde, advanced oxidation protein products, and systemic mitochondrial stress markers (including plasma mtDNA copies and lactate/pyruvate ratios) demonstrate partial correlations with reproductive oxidative status in certain cohorts. The necessity for ovary-proximal measurements in clinical and translational research is underscored by the persistence of granulosa-cell telomerase activity and follicular fluid markers as the most physiologically relevant indicators of ovarian ageing.

### 5.3. Evidence About the Conditions: POI, Endometriosis, and PCOS

PCOS is characterised by persistent low-grade inflammation, metabolic irregularities, and mitochondrial impairment in granulosa and cumulus cells. These factors elevate OS and accelerate telomere deterioration [96]. Prospective follicular studies indicate that oxidative stress-induced telomere/DDR phenotypes and premature somatic follicular ageing are associated with shorter telomeres in PCOS granulosa and cumulus cells, as well as dysregulation of shelterin components (TRF1 localisation in cumulus cells; altered TRF2 expression in both cumulus and granulosa cells) and the upregulation of pro-apoptotic signalling (BAX) [66]. These telomere modifications correspond with extensive studies on PCOS redox, linking telomere instability to aberrant folliculogenesis and diminished oocyte quality, emphasising increased ROS levels and compromised mitochondrial function in GCs [76]. Clinically, OS-telomere disruption provides a credible explanation for the ongoing difficulties encountered by PCOS patients in oocyte maturation and fertilisation, despite increased antral follicle numbers. It also supports the use of ovary-proximal telomere metrics (GC TL, TA) and follicular fluid oxidative indicators to assess the risk level for this phenotype [97].

Endometriosis maintains the ovarian microenvironment in a perpetual state of oxidative stress and inflammation. Granulosa cells from afflicted individuals exhibit senescence markers (↑ SA-β-gal), activation of endoplasmic reticulum stress, collapse of mitochondrial membrane potential, and depletion of ATP, leading to reduced oocyte production and competence [16]. Melatonin, which reduces endoplasmic reticulum stress, enhances mitochondrial function, and normalises GC/oocyte parameters both in vitro and in vivo, reverses the mechanisms that induce oxidative damage at telomeres, thereby halting replication forks and diminishing TRF1/TRF2 protection, resulting in the activation of the DNA damage response and cellular senescence [16]. Human IVF cohorts demonstrate that increased concentrations of 8-OHdG in follicular fluid and granulosa cells are associated with a decreased quantity of retrieved and mature oocytes, poorer fertilisation rates, and a reduced occurrence of blastocyst development. Moreover, the activity of GC telomerase declines with heightened oxidative damage, so demonstrating a clear correlation between oxidative stress and impaired telomere preservation, which leads to suboptimal IVF results [97]. These data suggest that redox-telomere damage is a key and modifiable factor leading to infertility linked to endometriosis.

POI refers to premature ovarian insufficiency. POI exemplifies a human model of accelerated reproductive ageing, marked by significant changes in telomere biology. A PRISMA-based study synthesising human data reveals that women with POI often have shorter telomeres and/or reduced telomerase activity in granulosa cells and/or leukocytes [30]. Nevertheless, methodological diversity and limited sample numbers diminish assurance. Despite recognising inconsistent leukocyte findings in certain populations and promoting standardised assays alongside more extensive, well-characterized studies, thorough reproductive reviews agree that telomere shortening is associated with diminished ovarian function [49,98]. Ultimately, the data indicates that telomere attrition and telomerase deficiency are components of the pathogenesis of POI, correlating with heightened oxidative stress, impaired DNA repair, and premature follicular depletion. A prevalent trend is seen among PCOS, endometriosis, and POI: Prolonged oxidative stress induces telomeric DNA damage (e.g., 8-OHdG), causing disruption of shelterin/DDR, cellular senescence, and mitochondrial dysfunction, which eventually diminishes oocyte competence [99]. The oxidative stress–telomere axis signifies the intersection of disease-specific modifiers, but with distinctions: metabolic-mitochondrial stress in PCOS, inflammatory/ER stress in endometriosis, and intrinsic telomerase/telomere abnormalities in POI. These findings highlight the significance of ovary-proximal measurements (GC/CC TL, TA; FF 8-OHdG) over peripheral leukocyte surrogates, and they promote disease-specific translational strategies, including metabolic/AMPK-SIRT1 modulation in PCOS, antioxidant/ER-stress reduction (melatonin) in endometriosis, and early telomere-informed counselling in POI [100]. Table 2 illustrates the distinctions among telomere-oxidative signatures in PCOS, endometriosis, and POI.

Collectively studied, the oxidative–telomeric phenotype demonstrates considerable diversity across PCOS, endometriosis, and POI, underscoring the primary metabolic stresses associated with each illness. Despite the increased quantity of antral follicles, hyperinsulinemia and excessive reactive oxygen species generation lead to accelerated ageing of somatic follicles in PCOS. This indicates that telomere disruption is mostly attributable to prolonged metabolic and mitochondrial stress. In contrast, endometriosis displays an inflammatory–ER-stress–induced telomere phenotype, marked by reduced mitochondrial potential and expedited granulosa-cell senescence resulting from continuous oxidative and inflammatory exposure. Various cohorts have shorter telomeres or reduced telomerase activity, regardless of extrinsic metabolic or inflammatory factors. Consequently, POI constitutes a unique category whereby telomere dysfunction may resemble a main deficiency. Thus, telomere dysfunction must be seen mechanistically as a hybrid phenomenon, first arising as a secondary effect of disease-specific stresses, and then, after the degradation of telomeric integrity, becoming a self-sustaining catalyst of defective folliculogenesis. Granulosa-cell telomerase activity, follicular-fluid 8-OHdG, and GC/CC telomere length are telomere-based assays that may be utilised in the future to classify disease subtypes, differentiate metabolic from inflammatory oxidative phenotypes, and predict treatment responses to antioxidant, mitochondrial, or metabolic therapies. Prior to the use of these tools for clinical decision-making, they must undergo standardisation and prospective validation.

### 5.4. Discrepancies in Evidence from Several Research

Despite growing evidence endorsing the oxidative stress telomere axis in reproductive ageing, not all human research exhibits a consistent association between telomere length and fertility outcomes. The disparities, mostly arising from variations in tissue type collected, assay used, patient demographics, and reproductive outcomes assessed, illustrate the challenges of integrating telomere biology into clinical practice [101]. Multiple comprehensive epidemiological studies investigating LTL demonstrate no correlation between telomere length and natural fecundity, pregnancy loss risk, or live birth rates in young to mid-reproductive age women attempting natural conception [24]. The Norwegian Mother, Father, and Child Cohort data also revealed no significant link between LTL and women’s probability of conception, infertility diagnosis, or the need for ART. However, an unexpected association was identified between prolonged male LTL and heightened ART utilisation, ascribed to behavioural and socioeconomic variables rather than biological deficit [87]. The findings indicate that, especially in younger individuals with mostly intact ovarian function, peripheral leukocyte telomere length may not effectively detect early reproductive decline.

Moreover, uneven repeatability arises from a lack of standardisation in methodologies among institutions, variations in sample preparation, and disparate techniques for assessing telomeres (qPCR, Southern blot, qFISH, and digital PCR). Due to financial and technological constraints, large studies often neglect to examine the temporal variations in telomerase activity, a component intricately associated with IVF results [102]. This may obscure telomere effects that are crucial for health when just static length is assessed. A significant concern is that it functions just on certain tissues. In folliculogenesis, granulosa and cumulus cells subjected to heightened oxidative and metabolic stress often have telomere patterns that differ from those of peripheral leukocytes. Investigations that provide null findings often rely on leukocyte evaluations, while IVF-related investigations using granulosa cells frequently demonstrate associations between telomere state and oocyte competence [103]. Peripheral telomeres are an insufficient replacement for ovarian telomere biology, which functions under unique metabolic, replicative, and oxidative challenges, as shown by conflicting findings.

Ultimately, there exists the matter of reproductive variety. In contrast to fertile younger women, telomere connections are reduced or nonexistent in instances of late maternal age, impaired ovarian reserve, and pathological oxidative stress situations such as PCOS and endometriosis. These differences suggest that telomere-based indicators may not be universally applicable across all reproductive ages, but are more pertinent in physiologically challenged ovarian contexts. In conclusion, the current disparities highlight the imperative for ovary-specific telomere evaluation, standardised methodology, and prospective assessment across clearly defined patient phenotypes, rather than challenging the biological plausibility of the oxidative stress telomere hypothesis in reproduction. To rectify these discrepancies and ascertain clinical significance, future studies must include telomerase activity, oxidative biomarkers, and multi-omics profiling in granulosa cells and follicular fluid, rather than just in leukocytes.

## 6. Methods for Evaluating the Axis

### 6.1. Methods for Determining Telomere Length

Multiple methodologies exist for quantifying TL, each with distinct advantages and disadvantages in the context of reproductive research. qPCR is the predominant technique used in IVF telomere investigations. It enables the simultaneous testing of several samples from minimal quantities of DNA, which is advantageous for precious ovarian specimens such as cumulus cells and granulosa cells [104]. The T/S ratio derived from qPCR enables the comparison of telomere lengths. This measurement may vary due to differences in batch, DNA quality, pipetting precision, and reference gene stability. To guarantee repeatability, it is essential to diligently include inter-plate controls, duplicate reactions, and calibrators [105]. Although qPCR is cost-effective and beneficial, it cannot differentiate between short dysfunctional telomeres and the overall telomere distribution, thereby failing to identify significant telomere attrition events that may contribute to reproductive ageing.

### 6.2. Evaluation of Telomerase Activity

TA is a dynamic indicator of cellular capacity to preserve telomeres. It demonstrates the efficacy of cells in preventing telomere shortening and repairing oxidative damage to telomeres. The Telomeric Repeat Amplification Protocol (TRAP) is the principal test used in reproductive research, sometimes applied in conjunction with fluorescence or quantitative PCR readouts to measure enzymatic extension products [106]. qPCR-TRAP works well with little quantities of ovarian cells obtained during IVF, since it is capable of detecting them in tiny samples. However, telomerase enzymes exhibit significant sensitivity to the composition of the buffer, proteolytic degradation, and freeze-thaw cycles. This indicates that they need prompt processing, meticulous handling, and dependable lysis reagents [95]. To guarantee assay precision and eradicate false-positive amplification, it is essential to validate the linear range, use heat-inactivated controls, and implement internal standards.

### 6.3. Biomarkers for Oxidative Stress

Assessing OS, a primary contributor to telomere deterioration and follicular impairment, is crucial for comprehending ovarian ageing. 8-hydroxy-2′-deoxyguanosine (8-OHdG) is the predominant biomarker for oxidative DNA damage, including in telomeric areas [14]. In ART research, it is practical and often used to do ELISA-based assessments in follicular fluid or granulosa-cell lysates; heightened levels are consistently correlated with a reduced number of retrieved oocytes, less mature MII oocytes, diminished fertilisation rates, and poorer blastocyst output. Owing to technological limitations, chromatographic systems with improved specificity, such as LC-MS/MS and HPLC, are not widely used [107]. Complementary biomarkers, such as protein carbonylation, lipid peroxidation products like malondialdehyde, total antioxidant capacity, and thiol-redox indices (GSH/GSSG), enhance oxidative profiling and aid in distinguishing between systemic and follicle-localized oxidative stress. Table 3 shows a list of the most important oxidative biomarkers found in FF/GCs and how they relate to clinical conditions.

Mitochondrial failure significantly contributes to ROS generation in the ovaries, making tests that target mitochondria especially pertinent. This includes ATP measurement, mitochondrial ROS fluorometry, mitochondrial membrane potential (ΔΨm) tests, and enzymatic activity profiling of antioxidant defences (e.g., superoxide dismutases, glutathione peroxidases, catalase) [108]. To appropriately interpret oxidative biomarkers, it is essential to monitor pre-analytical variables: the FF aspiration method, the extent of blood contamination, the time relative to the hCG trigger, and the storage temperature may significantly influence redox values. Standardising the collection of follicular fluid, either by sampling the first aspirated dominant follicle or using pooled standardised collection, enhances test comparability. To maintain methodological openness, research must publish recovery values, cross-reactivity controls, and assay variability, including both intra- and inter-assay coefficients [94]. The comprehensive assessment of robust oxidative biomarkers enhances telomere measures by elucidating the mechanisms of mitochondrial damage, cellular redox imbalance, and diminished oocyte competence in IVF cycles, while also identifying the factors contributing to telomere erosion.

### 6.4. Origins of Tissue and Fluid

The selection of biological substrate significantly influences the comprehensibility of telomere and oxidative stress measures. Granulosa and cumulus cells are the most physiologically significant substrates. They safeguard against oxidative stress during follicular development, provide metabolic substrates, and directly facilitate oocyte growth [109]. These cells function as superior biomarkers relative to peripheral blood, since their telomere length and telomerase activity demonstrate a robust association with oocyte cytoplasmic competence, meiotic quality, and fertilisation outcomes. FF offers substantial insight into the endocrine signalling framework, the intrafollicular redox milieu, and exposure to inflammatory or metabolic stresses [110]. The biochemical environment experienced by the developing oocyte is directly reflected by the measurement of oxidative stress indicators, antioxidant enzymes, metabolic intermediates, and inflammatory factors obtained by follicular fluid collection.

Leukocyte telomere length reflects systemic biological ageing but has contradictory associations with ovarian reserve, IVF response, and likelihood of conception. This disparity illustrates the variation in telomere regulation across different tissues and underscores the need of sampling near the ovaries for reproductive relevance [83]. It is essential to possess a valid rationale for using leukocyte analysis (for instance, an investigation of systemic ageing), and it is crucial to use caution when interpreting the data in relation to ovarian physiology. Seasonal, lifestyle, hormonal, and metabolic variables may have varying influences on systemic and ovarian telomere dynamics. Standardisation of sample handling for all tissue types is essential [41]. Isolation of granulosa and cumulus cells must ensure cellular purity, prevent RBC contamination, and record the stimulation methodology, follicle size characteristics, and processing length from retrieval. Whenever feasible, the most physiologically coherent dataset is generated by correlating granulosa or cumulus telomere length and telomerase activity with the oxidative indicators of the corresponding follicular fluid [111]. Two significant issues in the discipline are reproducibility and cross-study comparability. Disclosing methodological specifics such as needle gauge, suction pressure, medium composition, and procedure time helps mitigate these issues. The methodological variability in telomere biology research necessitates an understanding of the current assays and their limitations (Table 4).

The disparity in the methodologies used to assess telomere length and telomerase activity significantly contributes to the variability seen in published research. Due to its little DNA need and compatibility with granulosa or cumulus cells, qPCR is often used in reproductive research. Nonetheless, it provides only a relative T/S ratio and is very susceptible to batch effects, primer design, DNA quality, and inter-laboratory calibration. TRF Southern blotting, conversely, provides comprehensive length-distribution profiles and precise telomere measurements. However, it requires a substantial amount of high-quality DNA, which is seldom present in IVF samples. Single-cell methodologies such as qFISH and Flow-FISH may delineate telomere length within granulosa cell subpopulations and provide enhanced spatial resolution. Nonetheless, they are technically challenging, expensive, and difficult to standardise across facilities. The methodological discrepancies complicate the comparison of investigations; findings using qPCR often exhibit more variability and less repeatability compared to analysis employing TRF. Granulosa-cell telomerase activity, evaluated using qPCR-TRAP, now seems to be the most functionally significant biomarker for reproductive research. The inclusion of inter-plate reference controls, accurate calibration curves, and the recording of assay variability may improve qPCR-based telomere length tests. As the discipline advances, consensus guidelines for assay standardisation, standardised reporting standards, and cross-platform validation will be crucial to enhance reproducibility and facilitate direct comparisons across IVF cohorts.

## 7. Discussion

This analysis highlights a complicated and compelling interaction between telomere biology, oxidative stress, and reproductive outcomes in women undergoing assisted reproduction. The findings generally indicate that enhanced oocyte competence, embryonic development, and IVF success correlate with the integrity of intracellular telomeres and a reduced oxidative load in the follicular milieu. Peripheral circulating markers exhibit inconsistent associations, indicating that ovary-specific measures are crucial for obtaining physiologically relevant signals of reproductive ageing. Wang et al. (2014) performed a seminal research demonstrating that women who conceived after IVF had significantly increased TA in luteinized granulosa cells relative to those who did not (0.8825 vs. 0.513 OD × mm, *p* < 0.05) [47]. The lack of a notable variation in TL across the groups highlights the importance of functional telomerase competency over absolute telomere length in predicting reproductive success. Furthermore, with an odds ratio of 5.769 (95% CI: 1.434–23.212; *p* < 0.014), TA exceeded traditional indications such as age, basal FSH, and oestradiol levels as a significant independent predictor of conception. The predictive advantage was validated by ROC analysis (AUC 0.674 for TA compared to 0.576 for TL), indicating that telomerase activity is a potential biomarker [47].

A sequence of converging mechanistic principles defining the increasing agreement on the oxidative stress telomere axis in human reproduction can now be delineated, beyond a simply descriptive inventory of investigations. Three persistent themes arise from many clinical, translational, and disease-specific investigations: (1) Mitochondrial telomeric interactions create a self-perpetuating cycle in which ROS production, telomerase inhibition, and mitochondrial instability mutually amplify one another, (2) telomerase activity, rather than fixed telomere length, is the most sensitive functional marker of follicular biological age, and (3) pathological conditions such as PCOS, endometriosis, and POI exhibit unique yet mechanistically related oxidative telomeric profiles. These concepts converge to provide a definitive understanding of consensus: telomere integrity serves as an active regulatory hub that integrates mitochondrial function, metabolic status, and oxidative stress to ascertain oocyte competence, rather than just indicating ovarian ageing. The present evaluation provides a cohesive interpretation that integrates several findings into a singular biological model by selecting literature via this mechanistic lens.

Jiang et al. (2025), who investigated granulosa cell telomere length in 240 first-cycle IVF patients, concur with these findings [49]. Fertilisation rates (retrieved oocytes r = 0.408, *p* < 0.001; matured oocytes r = 0.203, *p* = 0.002) and TL showed a substantial positive connection with oocyte maturity (r = 0.386, *p* < 0.001). Although TL alone did not correlate with ovarian reserve parameters, ROC analysis revealed significant predictive value (AUC 0.719 for oocyte maturity). This suggests that oocyte competence and telomere dynamics are more closely correlated than the magnitude of response. Péntek et al. (2023) substantiated this by demonstrating that FF has less telomerase activity and markedly increased oxidative DNA damage (8-OHdG) in comparison to granulosa cells [41]. Granulosa cell 8-OHdG had a negative link with both GC and FF TA, while FF 8-OHdG levels were inversely related to recovered oocytes, MII count, fertilisation rate, and blastocyst development (*p* < 0.01). This suggests that oxidative telomere degradation may impair oocyte developmental ability by creating a mechanistic connection between oxidative stress, reduced telomerase activity, and decreased reproductive success.

The disease-specific patterns elucidate this axis further. Lamsira et al. (2024) indicate that patients with PCOS have dysregulated shelterin proteins, marked by heightened expression of TRF1/TRF2 and augmented BAX-mediated apoptosis, in addition to shorter telomeres in cumulus and granulosa cells [56]. The results validate the concept that oxidative stress linked to hyperandrogenism hastens telomere degradation and granulosa cell ageing in PCOS, possibly affecting folliculogenesis while preserving follicle quantity. Their results corroborate the evidence of mitochondrial malfunction and oxidative stress in PCOS [76]. Lin et al. (2020) showed that excessive oxidative stress in endometriosis inhibits oocyte maturation by inducing granulosa cell senescence, provoking endoplasmic reticulum stress, impairing mitochondria, and depleting ATP levels [27]. Melatonin rectified these alterations, indicating that antioxidant modulation may serve as a method to address issues with mitochondria and telomeres.

Research on natural fertility ageing clarifies the essential biology beyond disease conditions. Michaeli et al. (2022) discovered that women who conceived naturally between the ages of 43 and 48 had significantly longer leukocyte telomere length compared to their infertile counterparts of the same age (~9350 bp vs. 8850 bp; *p* = 0.03) [50]. This suggests that systemic telomere preservation may signal exceptional reproductive lifespan. In contrast, leukocyte telomere length showed no correlation with fecundability, pregnancy loss, or the use of assisted reproductive technology in younger women who conceived naturally, as evidenced by comprehensive prospective cohort studies by Purdue-Smithe et al. (2021) and Skåra et al. (2024) [38,95]. These data indicate that leukocyte telomere length may define extremes in reproductive longevity; nevertheless, it lacks the necessary precision for regular fertility prediction, especially in younger populations. Longo et al. (2024) also observed that granulosa-cell telomere length did not correspond with ovarian reserve or euploidy in the setting of IVF, but reduced leukocyte telomere length was associated with increased rates of embryo aneuploidy [94]. This suggests that leukocyte telomere length reflects systemic ageing effects on meiotic fidelity rather than follicular reserve. When integrated with the previously examined longitudinal and interventional data, these findings provide further support for a directed paradigm in which oxidative stress functions as a precursor to telomere malfunction, rather than as a simultaneous outcome of ovarian ageing [112]. Antioxidant or mitochondrial-targeted treatments partly mitigate telomeric attrition and rejuvenate telomerase activity. The experimental introduction of oxidative stress in animal models hastens telomere depletion in ovarian and somatic tissues. Human physiological investigations demonstrate that telomeric oxidative lesions occur before structural erosion, since oxidative damage at telomeric areas (8-OHdG accumulation) is apparent prior to detectable telomere shortening. Significantly, even if telomere length is constant, telomerase activity in granulosa cells rapidly declines in the presence of oxidative stress. This corroborates the notion that functional telomere dysfunction occurs at an earlier biological phase. These data indicate a sequence of events whereby oxidative stress compromises meiotic integrity, accelerates ovarian biological ageing, and destabilises the telomere maintenance mechanism.

### 7.1. Biological Interpretation & Mechanistic Integration

Evidence from cellular, biochemical, and clinical studies suggests a common mechanism in which oxidative stress impairs granulosa-oocyte function and reproductive capability by hastening telomere degradation and hindering telomerase-mediated genomic protection [20]. Telomeres rich in guanine repeats are particularly susceptible to oxidative damage from ROS, such as 8-hydroxy-2-deoxyguanosine, which has been detected in follicular fluid at significantly elevated levels compared to granulosa cells [103]. Oxidative DNA damage induces replication fork stalling, telomere instability, and the activation of DNA damage response signalling. This pattern indicates that the follicular microenvironment functions as both a site of conflict and a reservoir for oxidative damage [113].

Essential shelterin proteins, including as TRF1 and TRF2, govern replication and protect telomeres. Clinical findings demonstrate that PCOS is associated with aberrant TRF1/2 expression, implying stress-induced compromise of telomere structural integrity [66]. Specifically, the depletion of TRF2 disrupts t-loop formation and compromises telomere end protection, rendering telomeres susceptible to nucleolytic assault and resulting in telomere uncapping. These alterations have been associated with oocyte developmental failure and accelerated apoptosis in granulosa cells, shown by elevated BAX expression. The buildup of ROS inflicts damage on telomeres at the mitochondrial level, establishing a feedback loop where telomere dysfunction hinders mitochondrial biogenesis via p53–PGC-1α signalling, while mitochondrial dysfunction exacerbates ROS generation. Melatonin treatment may counteract this reciprocal degradation, leading to reduced ATP generation, as shown in GC senescence linked to endometriosis [16]. SIRT1, a crucial NAD^+^-dependent deacetylase that safeguards telomeres and enhances mitochondrial resilience, fortifies the telomere–mitochondria–sirtuin axis in reproductive ageing [114].

Telomerase serves as the convergence point for all these routes. It not only extends telomeres but also stabilises the DNA, remodels chromatin, and safeguards against oxidative stress. Research conducted by Wang et al. (2014) and Péntek et al. (2023) demonstrates that telomerase activity, as opposed to fixed telomere length, more precisely indicates oocyte competence and the effectiveness of IVF [41,107]. Increased TA significantly improves the probability of conception after IVF by roughly five times, in addition to its correlation with pregnancy outcomes. This indicates that telomerase is not only a passive enzyme that elongates telomeres. It also seems to function as a dynamic molecular rheostat that protects cells against oxidative stress. Moreover, the linkages between the immunological and endocrine systems seem to be substantial. Increased leukocyte telomere length in women who achieve spontaneous conception in their late 40 s indicates systemic genomic resilience in extraordinary female reproductive characteristics [35]. Nonetheless, extensive cohort studies demonstrate that leukocyte telomere length in healthy reproductive-age women lacks predictive value, thereby confirming tissue-specific telomere biology and highlighting the importance of ovarian-proximal measurements (GCs, follicular fluid) [24,87].

### 7.2. Interpretation of Conflicting Findings and Sources of Variability

Our research is varied, with considerable variability across study populations, measurement methodologies, and biological compartments, despite a growing agreement that oxidative stress and telomere disruption are pivotal factors in ovarian ageing and IVF results. These apparent discrepancies highlight the complexity of reproductive ageing and the necessity of contextualising biomarkers within tissue specificity, reproductive phenotype, assessment timing, and environmental exposures, rather than undermining the biological significance of the oxidative stress telomere axis. The biological basis of telomere assessment is a significant source of variability. Extensive prospective cohorts by Purdue-Smithe et al. (2021) and Skåra et al. (2024) demonstrate that investigations of leukocyte telomere length (LTL) often produce neutral or modest associations with fecundability or IVF results [38,95]. The results demonstrate that in women of reproductive age, systemic telomere attrition does not precisely represent the local ovarian microenvironment; yet, it does signify biological ageing on a broader level. Wang et al. (2014) and Jiang et al. (2025) have shown that telomere metrics obtained from granulosa cells provide much superior prediction value for oocyte maturation and fertilisation potential [47,49]. This confirms that ovarian-proximal measures properly represent real-time cellular ageing processes, including mitochondrial functionality, oxidative stress, and telomere protection capability.

The age distribution of the research cohorts significantly influences the observed relationships. At physiological extremes, the connections between telomeres and reproduction become more evident. Women who conceived naturally between the ages of 43 and 48 demonstrated markedly longer leukocyte telomere length (LTL) than age-matched infertile controls indicating that systemic telomere resilience may correlate with exceptional reproductive longevity phenotypes [35]. Purdue-Smithe et al. (2021) contend that groups of younger women with comparable ovarian reserves may not exhibit a significant biological gradient to identify differences [38]. Thus, telomere-based indicators may be used to distinguish between rapid and extraordinary reproductive ageing trajectories instead of functioning as generic predictors. The third axis of heterogeneity pertains to disease-specific telomere pathology. Telomere biology in PCOS exhibits a contradiction, marked by shorter telomeres and aberrant expression of the shelterin proteins (TRF1/TRF2) in granulosa and cumulus cells, while maintaining ovarian reserve [66]. Oxidative stress, persistent hyperinsulinemia, and hyperandrogenism are the probable aetiologies of these diseases [76]. Granulosa-cell senescence and mitochondrial dysfunction mediated by oxidative stress are the primary contributors of endometriosis [16]. Nonetheless, systemic telomere measurements vary throughout different investigations. This intricacy warns against simplistic linear models by demonstrating the complicated relationships among mitochondrial function, telomere control, metabolic signalling, and inflammation.

Methodological variability exacerbates disparities. Various approaches for quantifying TL, including as qPCR, monochrome multiplex qPCR, qFISH, and Southern blot TRF tests, exhibit differing degrees of sensitivity, cellular resolution, and vulnerability to oxidative degradation. TRF analysis exhibits more accuracy; nonetheless, it is ineffective for large cohorts. Quantitative PCR-based telomere length estimations are often used in reproductive research; nevertheless, they may be influenced by pre-analytical variability, mitochondrial DNA content, and the composition of various leukocyte types [115]. The analytical precision and dynamic range of telomerase activity tests (PCR-TRAP, fluorescence-based TRAP, and qPCR-based TA quantification) demonstrate considerable disparity. The difference in predictive value between TL and TA highlights the significance of functional telomere-maintenance ability over mere telomere length, as shown by Péntek et al. (2023) and Wang et al. (2014) [41,107]. Environmental and temporal considerations provide an additional significant layer of complexity. Reviews on oxidative reproductive ageing and metabolic telomere research suggest that telomere dynamics are heterogeneous and influenced by oxidative stress, inflammation, metabolic strain, glucocorticoid signalling, and lifestyle factors including BMI, smoking, and glycaemic load [41,45]. Thus, telomere measures obtained at a singular time point may inadequately represent the real-time adaptive biochemical robustness of the ovarian microenvironment.

### 7.3. Clinical Implications for Assisted Reproduction

The growing research linking oxidative stress, telomere malfunction, and reproductive ageing will significantly impact clinical reproductive practice. Although conventional reproductive indicators including antral follicle count, FSH, AMH, and oestradiol are essential for assessing ovarian reserve, they mostly reflect gamete number rather than cellular quality, genetic stability, and metabolic resilience. The domain must evolve towards a more complex, molecularly informed assessment of ovarian function, since there is a growing recognition that oxidative stress hastens telomere degradation in granulosa and cumulus cells, thereby reducing oocyte competence.

A recurring theme in the examined research is that telomere integrity and telomerase activity are indicative of mitochondrial function, follicular cell vitality, and resilience to oxidative stress. In clinical IVF populations, granulosa-cell telomerase activity has consistently shown more predictive ability than telomere length or traditional endocrine indicators. Wang et al. demonstrated that granulosa-cell telomerase activity, rather than telomere length, was a significant predictor of clinical pregnancy after IVF [47]. Women exhibiting elevated enzymatic activity had about fivefold the likelihood of achieving pregnancy. Age and starting hormone levels did not affect this prediction capacity, highlighting that telomerase activity functions as a physiologically active indicator of continuous genomic defence rather than just a record of past replication history [80]. The findings indicate that for certain patients, especially those with recurrent fertilisation failure, unexpected poor oocyte performance, or unexplained subfertility, assessing granulosa-cell telomerase activity may improve prognostic precision and enable the personalisation of treatment approaches.

The oxidative stress telomere axis is receiving growing endorsement. Its therapeutic applicability is impeded by substantial information gaps. There is a lack of long-term human research that may clarify temporal causation, especially about whether telomere dysfunction precedes, corresponds with, or follows the earliest phases of follicular decline. Secondly, comparing outcomes across reproductive cohorts is challenging because to the variability in telomere testing techniques and the lack of assay standardisation. Third, the molecular relationships between telomerase activity, mitochondrial malfunction, and replication stress are little understood, especially in the context of healthy vs. pathological ageing. Fourth, although follicular oxidative indicators and granulosa-cell telomerase activity seem promising as possibilities, no telomere-based biomarker has been validated for predicting an individual’s response to stimulation procedures or for the early diagnosis of preclinical ovarian ageing. Ultimately, there is less knowledge on the optimal timing, dosage, and potential long-term impacts on germline integrity. The safety and efficacy of telomerase-targeted or mitochondrial treatments remain largely unverified. The main obstacle in converting the oxidative stress–telomere axis into therapeutically viable tools is overcoming these information gaps.

## 8. Strengths and Limitations of the Evidence

The current corpus of research on the oxidative stress-telomere axis in female reproduction offers several substantial advantages. Numerous independent clinical studies involving diverse patient populations, including IVF cohorts, naturally fertile women of advanced maternal age, and individuals with reproductive disorders such as PCOS and endometriosis, consistently reveal mechanistic and clinical correlations between oxidative stress, telomere integrity, and reproductive potential. Granulosa cells and follicular fluid represent the most physiologically informative tissues in human reproductive research, as shown by studies conducted by Wang et al., Péntek et al., and Jiang et al., which directly assess telomerase activity or telomere length [41,47,49]. These investigations provide prospective, therapeutically relevant data. These conclusions are supported by translational research indicating that antioxidant interventions, such as melatonin, can reverse telomere-related cellular dysfunction in conditions like endometriosis, along with mechanistic cellular studies revealing oxidative-mediated telomeric DNA damage, mitochondrial dysfunction, and granulosa cell senescence. These findings endorse a physiologically credible and practically relevant paradigm whereby oxidative stress hastens telomere erosion and impairs telomerase activity, eventually leading to oocyte deterioration and unsatisfactory reproductive consequences.

Notwithstanding these advantages, certain constraints temper the interpretation of the existing data. A notable constraint is the variability of methodological methodologies used to evaluate telomere biology in various investigations. Quantitative PCR, Southern blotting, qFISH, and PCR-based telomerase tests vary significantly in sensitivity, resolution, and repeatability. This complicates the comparison of research. Furthermore, leukocyte telomere length functions as a biomarker in other investigations. Despite its accessibility, it is inadequate as a substitute for ovarian cellular telomere dynamics and is influenced by systemic and ambient inflammatory variables. Conversely, measures from granulosa cells are only obtainable in IVF contexts and need intrusive sample, rendering them impractical for alternative therapeutic applications and long-term monitoring.

The study’s methodology and sample size remain significant issues. Many studies in this field use cross-sectional designs and have small participant numbers, which restricts statistical power to identify subtle relationships and hinders causal inference, especially in subgroup analyses based on age, metabolic state, or ovarian phenotype. Furthermore, other subtypes of reproductive phenotypes, including PCOS and endometriosis, exist; nevertheless, telomere-related research has seldom recognised this biological variety. This indicates the possibility of insufficient mechanistic characterisation and phenotypic dilution. A restricted number of research thoroughly examine the confounding factors affecting telomere length and oxidative stress, such as body mass index, smoking, dietary practices, sleep patterns, psychological stress, and environmental exposures. All of these variables may influence the ageing of reproductive cells.

A significant issue is the scarcity of longitudinal research. The majority of currently available data examine telomere status at a certain time point. This indicates that we are unable to ascertain the telomere attrition rate or the variations in oxidative stress during several IVF cycles or the reproductive lifetime. Ultimately, whereas animal models demonstrate the protective effectiveness of antioxidant and mitochondrial therapy, there is a lack of human interventional studies explicitly focused on telomere preservation in reproductive settings. Moreover, enduring safety issues, especially for telomerase-modulating drugs, need careful examination.

## 9. Conclusions

The information collected in this review demonstrates that the oxidative stress–telomere axis represents a crucial biological mechanism linked to ovarian ageing, diminished oocyte quality, and reduced reproductive capacity. The telomeres and telomerase activity of granulosa and cumulus cells are essential for maintaining genomic integrity throughout folliculogenesis and oocyte maturation. Moreover, growing data suggests that oxidative stress-induced telomere shortening and telomerase suppression compromise meiotic competence, fertilisation capacity, and early embryonic development. Clinical studies in IVF environments demonstrate that leukocyte-derived measurements and traditional endocrine indicators are less effective predictors of oocyte competence and reproductive outcomes than ovarian-proximal telomere and telomerase metrics, especially granulosa-cell telomerase activity. These data corroborate the idea that telomere biology mirrors the aggregate metabolic, oxidative, and mitochondrial stress encountered by the ovarian microenvironment, in conjunction with chronological ageing.

However, many methodological and translational shortcomings remain. Generalisability is currently limited by the diversity of patient characteristics, small sample numbers, variability in analytical methods, and the prevalence of cross-sectional designs. Mechanistic studies and preliminary clinical evidence indicate that therapies supporting antioxidants, metabolism, and mitochondrial function may mitigate telomere stress and enhance reproductive results. Nonetheless, rigorous interventional studies are needed to confirm effectiveness and safety, especially with telomerase-targeting approaches. The existing collection of research highlights the need of including molecular indicators of cellular ageing into reproductive assessment frameworks. Telomere biology enhances conventional techniques for assessing ovarian reserve by providing additional insights into the health and biological integrity of the follicular compartment. With the advancement of standardised telomere and oxidative stress tests, longitudinal datasets, and tailored therapy strategies, the oxidative stress–telomere axis may become a significant tool in clinical and research settings. This may provide novel approaches to enhance reproductive results, including the customisation of fertility treatments, improved forecasts of reproductive health, and more methodologies.

## Figures and Tables

**Table 1:** Human studies on telomeres/telomerase in IVF.

Study	Population	Tissue	Biomarkers	Key Findings	Clinical Relevance
Wang et al., 2025, [33]	IVF patients	Granulosa cells	Telomerase Activity (TA) & TL	↑ TA in pregnant vs. non-pregnant; TA AUC 0.674 vs. TL AUC 0.576; 5-fold ↑ pregnancy odds per TA unit	TA stronger predictor of IVF success than TL & age
Jiang et al., 2025, [49]	240 IVF patients	Granulosa cells	TL	TL correlated with fertilization (r = 0.408) & MII rate (r = 0.386); no association with blastulation	TL predicts oocyte maturity, not blastocyst stage
Péntek et al., 2023, [41]	102 IVF patients	FF & GCs	8-OHdG, TA	↑ 8-OHdG = ↓ oocytes, ↓ MII, ↓ fertilization, ↑ blastocysts; ↑ OS = ↓ TA	Oxidative DNA damage impairs telomere maintenance & IVF outcomes
Limonad et al., 2021, [59]	IVF patients	Granulosa cells	TL, metabolic markers	Longer TL with healthier FF metabolism; ↑ pregnancy (88% vs. 38%)	Metabolic health linked to TL & fertility
Longo et al., 2024, [94]	IVF/PGT patients	Leukocytes	TL	Short LTL associated with ↑ aneuploidy	Systemic TL may reflect chromosomal integrity risk
Purdue-Smithe et al., 2021, [38]	Natural conception	Leukocytes	TL	No association with fecundability or miscarriage	LTL weak biomarker in young fertile women
Skåra et al., 2024, [95]	Population cohort	Leukocytes	TL	No association LTL & female fertility/ART need	LTL not reliable marker for reproductive potential

Table 1: Human studies evaluating oxidative indicators in follicular fluid, telomere length and telomerase activity in granulosa or cumulus cells, and results of IVF (maturation, fertilisation, blastulation, and clinical pregnancy).

**Table 2:** Disease-specific telomere–oxidative stress patterns.

Condition	Telomere Findings	Oxidative Phenotype	Mechanistic Signature	Clinical Implication
PCOS	Shorter GC/CC TL; altered TRF1/TRF2	↑ ROS, mitochondrial dysfunction	Metabolic stress, insulin resistance, apoptosis	Telomere-metabolic phenotype despite high AFC
Endometriosis	GC senescence, ↓ TA	↑ FF oxidative load, ER stress	mt dysfunction, ATP loss, apoptosis	Melatonin improves telomere & oocyte quality
POI	Short TL, ↓ TA	↑ OS, DNA repair failure	Accelerated telomere exhaustion	Early biomarker for ovarian aging
Age-related decline	TL attrition in GCs; ↓ TA	Progressive oxidative burden	Loss of telomeric stability → spindle errors	Biological aging > chronological age
Normal fertility	Preserved TL, TA	Controlled ROS	Efficient antioxidant response	Maintains oocyte competence

Table 2: Oxidative stress and telomere profiles associated with female reproductive disorders. The unique telomere dysfunction mechanisms present in PCOS, endometriosis, and premature ovarian insufficiency are driven by metabolic, inflammatory, and mitochondrial stress pathways.

**Table 3:** Oxidative stress biomarkers in follicular fluid & granulosa cells and reproductive outcomes.

Biomarker	Biological Source	Analytical Method	Clinical Finding	Interpretation
8-OHdG	FF, GCs	ELISA/HPLC	↑ 8-OHdG = ↓ oocytes, MII, fertilization, blastulation	Direct oxidative DNA damage correlates with oocyte competence
MDA (malondialdehyde)	FF	Spectrophotometric	Increased in poor responders and PCOS	Lipid peroxidation contributes to follicular stress
TAC (Total Antioxidant Capacity)	FF	Colorimetric	Lower in endometriosis, poor response	Reduced antioxidant defense impairs oocyte maturation
SOD, GPx, Catalase	GCs, FF	Enzyme assays	↓ Antioxidant enzymes = impaired IVF outcomes	Antioxidative enzyme depletion affects COC health
mtROS	GCs	Fluorescent assays	↑ mtROS in PCOS/endometriosis GCs	Mitochondrial dysfunction → telomere erosion
mtDNA copy number	FF/GCs	qPCR	Altered in aging/PCOS	Reflects metabolic reserve & oxidative burden

Table 3 shows oxidative stress biomarkers (like 8-OHdG, TAC, SOD/GPx/catalase, and mtROS) that are found in granulosa cells and follicular fluid. It also shows how these biomarkers are related to oocyte maturation, fertilisation, blastocyst formation, and pregnancy rates.

**Table 4:** Laboratory methods for telomere and oxidative stress assessment.

Method	Parameter	Strengths	Limitations	Use in Reproductive Studies
qPCR TL (T/S ratio)	Relative TL	Low sample need, fast	Cannot detect critically short telomeres	Main method in IVF TL studies
TRF Southern blot	Absolute TL	Gold standard, length distribution	Requires high DNA qty, laborious	Limited use in ovarian tissue
qFISH/Flow-FISH	Single-cell TL	Cell-specific resolution	Expensive, technical	Useful in GC and CC telomere mapping
TRAP assay	Telomerase activity	Functional readout	Sensitive to handling	Best predictor of IVF success (GCs)
ELISA for 8-OHdG	Oxidative DNA damage	Easy, widely available	Possible cross-reactivity	Most common OS biomarker in FF
LC-MS/HPLC	8-OHdG, MDA	High specificity	Not routine in clinics	Research-level validation
JC-1, mtROS fluorometry	Mitochondrial redox	Functional mitochondrial readout	Technique-dependent	PCOS/Endometriosis mechanistic studies

Table 4: A compilation of methods for assessing oxidative stress, telomerase activity, and telomere length in reproductive studies. The understanding of telomere oxidative stress interactions in IVF depends on the specificity, technical complexity, and biological resolution of each approach.

## Data Availability

No new data were created or analyzed in this study. Data sharing is not applicable to this article.
